# Clinical Characteristics and Serum Cytokines Profiling in Hospitalized COVID-19 Patients in Lebanon

**DOI:** 10.1155/2023/7258585

**Published:** 2023-05-16

**Authors:** Farouk F. Abou Hassan, Mirna Bou Hamdan, Nada M. Melhem

**Affiliations:** ^1^Medical Laboratory Sciences Program, Division of Health Professions, Faculty of Health Sciences, American University of Beirut, Beirut, Lebanon; ^2^Center for Infectious Diseases Research, Faculty of Medicine, American University of Beirut, Beirut, Lebanon

## Abstract

Since its emergence, severe acute respiratory syndrome coronavirus 2 (SARS-CoV-2) remains a public health threat worldwide. While the majority of patients recover in 3–4 weeks, complications in severely ill patients, including acute respiratory distress syndrome, cardiac injury, thrombosis, and sepsis, can lead to death. Several biomarkers, in addition to cytokine release syndrome (CRS), have been associated with severe and fatal outcomes in coronavirus disease 2019 (COVID-19) patients. The aim of this study is to assess clinical characteristics and cytokines profiles in hospitalized COVID-19 patients in Lebanon. A total of 51 hospitalized COVID-19 patients were recruited between February 2021 and May 2022. Clinical data and sera were collected at two time points: at hospital presentation (T0) and last collected results during hospitalization (T1). Our results showed that 49% of participants were >60 years with males accounting for the majority (72.5%). Hypertension, followed by diabetes and dyslipidemia, were the most frequent comorbid conditions among study participants accounting for 56.9% and 31.4%, respectively. Chronic obstructive pulmonary disease (COPD) was the only significantly different comorbid condition between intensive care unit (ICU) and non-ICU patients. Our results also showed that the median level of D-dimer was significantly elevated among patients in ICU and those who died compared to non-ICU patients and those who survived. Moreover, C-reactive protein (CRP) levels were significantly higher at T0 compared to T1 in ICU and non-ICU patients. The median level of IL-12p70 was significantly higher in patients >60 years compared to those ≤60 years (*p* = 0.0209). Our data are in agreement with previous reports suggesting the importance of IL-6, CRP, and IL-12p70 in the assessment of risk of severe disease and mortality.

## 1. Introduction

Since its emergence in December 2019, severe acute respiratory syndrome coronavirus 2 (SARS-CoV-2), the causative agent of coronavirus disease 2019 (COVID-19), remains a global public health threat. COVID-19 causes a wide range of clinical manifestations ranging from asymptomatic to severe illness and death [1]. In symptomatic individuals, fever, cough, and shortness of breath are the most commonly reported symptoms; these symptoms can be accompanied by fatigue, myalgia, gastrointestinal symptoms (nausea, vomiting, diarrhea), headache, weakness, rhinorrhea, anosmia, and ageusia [1, 2]. The majority of patients recover in 3–4 weeks [3]. A number of complications have been reported in severely ill patients leading to COVID-19-related deaths; these include pneumonia, acute respiratory distress syndrome (ARDS), liver injury, cardiac injury, thrombosis, including stroke, renal disease, neurologic disease, and sepsis [1, 2]. Older age, male gender, and preexisting medical conditions, such as hypertension, diabetes, obesity, allergy and asthma, chronic obstructive pulmonary disease (COPD), interstitial lung disease, chronic liver diseases (CLD), chronic kidney diseases, cancer, and immunodeficiency, are risk factors for severe illness [4]. Few patients experience postacute COVID-19 syndrome, whereby patients suffer from a range of lingering COVID-19-related symptoms that can affect a number of organ systems and may last for weeks or months [3, 5].

Cytokine release syndrome (CRS) is a collection of clinical manifestations resulting from a life-threatening systemic inflammatory syndrome characterized by elevated levels of circulating cytokines and immune-cell hyperactivation [6, 7]. CRS was reported among patients with COVID-19; the former is characterized by increased levels of pro-inflammatory cytokines correlating with disease severity; these cytokines include interleukin (IL)-1*β*, IL-2, IL-6, IL-10, interferon (IFN)-*γ*, tumor necrosis factor (TNF), IFN-*γ*-inducible protein 10 (IP-10), granulocyte macrophage-colony stimulating factor (GM-CSF), and monocyte chemoattractant protein-1 (MCP-1) [8–12]. Particularly, high levels of serum IL-1, IL-6, IL-8, TNF, as well as C-reactive protein (CRP) at presentation, were strong predictors of disease severity and patients' survival [8, 12, 13]. These inflammatory mediators result in an influx of immune cells (macrophages, neutrophils, and T cells) to the site of inflammation leading to tissue damage, multiorgan failure, and death [13]. TNF mostly triggers apoptosis and necrosis in sensitive tissues, and excessive secretion of TNF is a key factor in the pathological development and can induce the development of several diseases [14, 15]; TNF also plays an important role during SARS-CoV-2 infection as it is upregulated in acute lung injury, triggers CRS and facilitates SARS-CoV-2 interaction with angiotensin–converting enzyme 2 (ACE2) [16]. Importantly, CRS may play a role in postacute sequelae of COVID-19. Elevated levels of plasma IL-1*β*, IL-6, and TNF were shown to be associated with postacute sequelae of COVID-19 [17].

High levels of cytokines (IL-2, IL-4, IL-6, IL-7, IL-8, IL-10, G-CSF, IP10, MCP-1, MIP-1A, TNF, and IFN-*γ*) were detected in sera of patients with severe COVID-19 illness [9, 18, 19]. Evidence suggests the possible role of pro-inflammatory cytokines in the pathogenesis of COVID-19 and related complications [20, 21]. These pro-inflammatory cytokines mainly include IL-1, IL-6, TNF, and MIP-1A [22]. Specifically, a high level of IL-6 was found to be associated with disease progression and respiratory failure in severe cases [23–25]. Other biomarkers, including CRP, procalcitonin (PCT), ferritin, and D-dimer, were also important prognostic indicators correlated with in-hospital mortality [25]. Several studies highlighted the potential role of D-dimer as a predictive biomarker for poor prognosis in hospitalized COVID-19 patients [26–28]. Few studies in the Eastern Mediterranean Region reported higher serum levels of IL-1*β*, IL-6, IL-8, and TNF in severe COVID-19 patients compared to mild cases [29]. Moreover, the expression level of genes encoding for IFN-*γ*, IL-2, IL-4, IL-6, IL-17, TGF-B, IL-8, and IL-1*β* was significantly higher in hospitalized COVID-19 patients compared to healthy controls; however, there was no significant difference in the expression level between intensive care unit (ICU)-admitted and non-ICU admitted patients in reported data [30].

The aim of our study is to compare the levels of cytokines among ICU, and non-ICU admitted patients following SARS-CoV-2 infection in a cohort of hospitalized patients in Lebanon.

## 2. Methods

### 2.1. Study Design, Population, and Data Collection

This study was approved by the Biomedical Institutional Review Board at the American University of Beirut (AUB). All participants signed a written informed consent. A total of 51 hospitalized COVID-19 patients (≥18 years) were recruited between February 2021 and May 2022 at the American University of Beirut Medical Center (AUBMC). During that time, the Pfizer-BioNTech COVID-19 vaccine (BNT162b2) was the main vaccine widely available in Lebanon; the Oxford-AstraZeneca (ChAdOx1) and Johnson & Johnson (Ad26.COV2) vaccines were also administered, albeit less frequently [31]. The BNT162b2 was the first vaccine to be delivered to Lebanon. The administration of the vaccine was prioritized into four phases to target high-risk individuals based on the risk of exposure and infection, risk of complications following infection, essential personnel (front-line responders to the pandemic, workers in primary healthcare centers), and the availability of vaccines [31]. During the first phase, the vaccine was administered to healthcare workers and all individuals ≥75 years old (Phase IA), followed by all individuals 65–74 years old, all individuals 55–64 years with comorbidities, and individuals working in COVID-19 epidemiological surveillance (Phase IB) [31]; the remaining phases included individuals 16–54 years with comorbidities (Phase IIA), essential personnel in the public sector and those working in nursing homes in addition to inmates with special needs (Phase IIB), teachers and workers in areas with a high risk of infection and people who care for elderly (Phase III), and finally all individuals willing to receive the vaccine (Phase IV). Prioritizing COVID-19 vaccine doses in Lebanon was in accordance with the recommendations of the WHO Strategic Advisory Group of Experts (SAGE) framework for the allocation and prioritization of COVID-19 vaccination in targeting high-risk groups at different stages of vaccine supply availability [32]. Our patients were recruited during the circulation of the Alpha (January–April 2021), Delta (June–July 2021), and Omicron (December 2021–May 2022) variants of concern [33, 34]. We categorized our study participants into two groups, as previously described [35]. The mild/moderate group included patients who did not require ICU admission, whereas the severe group included those requiring ICU admissions. Demographic and clinical data were collected from the medical records of hospitalized COVID-19 patients. Data were collected at two time points: at hospital presentation (T0) and last results during hospitalization (T1) (i.e., discharge or death). These data include age, sex, smoking history, preexisting comorbid conditions, COVID-19 vaccination status, date of COVID-19 testing, history of SARS-CoV-2 exposure, signs, and symptoms at presentation and during hospitalization, ICU admission, length of hospitalization/ICU admission until discharge or death, oxygen requirement, chest X-ray (CXR) and CT-scan reports, and list of medications. Moreover, data on hematological parameters were collected; these included a complete blood count, which was performed as part of the clinical care during hospitalization. We also collected the levels of CRP, D-dimer, and PCT from the medical charts, when available.

### 2.2. Cytokines Assay

Sera were only available for 23 hospitalized COVID-19 patients. Collected samples were stored at −80°C until processed. The levels of 11 cytokines (GM-CSF, IFN-*γ*, IL-1*β*, IL-2, IL-4, IL-5, IL-6, IL-12p70, IL-13, IL-18, and TNF) were measured using a commercially available Th1/Th2 Cytokine 11-Plex Human ProcartaPlex™ Panel (Thermo Fisher Scientific, USA; cat#: EPX110-10810-901; LOT: 253603-002) according to the manufacturer's instructions. Samples were analyzed in duplicates using the Luminex xMAP instrument (Software: xPonent v.4.2). The concentration of the cytokines was calculated using the standard curve generated by the 5-parameter logistic regression method on the online ProcartaPlex Analysis App (Thermo Fisher Scientific). We excluded from the analysis values that were out of range (OOR).

### 2.3. Statistical Analysis

Data were summarized descriptively using counts and frequencies for categorical variables and mean, standard deviation, and range for continuous variables. Fisher's exact test was used to investigate the association between qualitative variables (gender, hospitalization, comorbid conditions, and COVID-19 complications). We used the nonparametric Mann–Whitney *U*-test or Kruskal–Wallis test to compare the levels of cytokines between groups as appropriate. Paired data were analyzed by Wilcoxon signed rank test. A *p* value < 0.05 was considered significant. All analyses were performed using STATA SE 13.0. Figures were generated using GraphPad Prism 9.3.1.

## 3. Results

### 3.1. Demographic and Clinical Characteristics of Patients

A total of 51 hospitalized COVID-19 patients were included in this study, with almost half of the former being >60 years old. The majority of our participants were males (72.5%) and suffered from at least one comorbid condition (80%) (Table 1). The majority of hospitalized patients (70.5%) were not vaccinated against COVID-19. Four participants only (7.8%) received ≥2 doses of the vaccine, while vaccination data were not available on the remaining participants (*n* = 11; 21.5%). Hypertension, followed by diabetes and dyslipidemia, were the most frequent comorbid conditions among study participants accounting for 56.9% and 31.4%, respectively. Upon hospitalization and as documented in the medical charts of our study participants, the most commonly reported symptoms at presentation were fever (72.5%), followed by cough (66.7%) and dyspnea (66.7%). Supplementary oxygen was needed for 35 patients (70%). Out of the 51 hospitalized patients, 11 (21.6%) were admitted to the ICU. CXR and CT-scan findings at the presentation were available for 28 (54.9%) and 44 (86.3%) patients, respectively. While the CXR results showed diffuse bilateral interstitial and airspace opacities in 19 patients, the chest CT-scan showed severe pneumonia in 11 patients, out of which four patients were admitted to the ICU. A total of nine patients died during hospitalization (median age 81 years); six out of the former were in ICU and needed mechanical ventilation (Table 1). Most deaths (88.9%; *n* = 8) were among patients >60 years compared to one death of a patient <60 years old (*p* = 0.023); moreover, the majority of patients who died (66.7%; *n* = 6) had hypertension (data not shown). The medical records did not reveal any data on COVID-19 vaccination for deceased patients.

The median age of ICU-admitted patients was 70 years compared to 57.5 years among non-ICU admitted patients (*p* = 0.0587) (Table 2). All ICU-admitted patients were males (*p* = 0.023). COPD was the only significantly different comorbid condition between ICU and non-ICU patients (*p* = 0.006). As expected, all ICU patients required supplementary oxygen compared to 61.5% of those who were not admitted (*p* = 0.021); none of the latter required mechanical ventilation. The median duration of supplementary oxygen requirement was longer among ICU patients. Moreover, 63.6% (*n* = 7) and 54.6% (*n* = 6) of ICU-admitted patients were treated with CRS and IL-6 receptor antagonist treatments, respectively, compared to 12.5% (*n* = 5) and 15.4% (*n* = 6) of patients who did not require ICU admission (Table 2). We did not detect any significant difference in CXR and CT scan findings between the two groups (data not shown). More than 50% of patients who were admitted to the ICU died compared to 7.9% of those who were not (*p* = 0.002). Among those patients who were admitted to the ICU and died with available chest CT-scan results (*n* = 8), 62.5% (*n* = 5) had severe pneumonia compared to 12.5% (*n* = 1) with mild/moderate pneumonia (*p* = 0.002) (data not shown).

### 3.2. Laboratory Biomarkers and Cytokines Measurement

**Figure 1:**
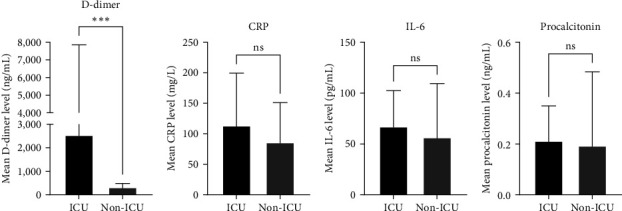
Levels of clinical parameters among ICU and non-ICU admitted patients. Data on D-dimer (a), CRP (b), IL-6 (c), and procalcitonin (d) were extracted from the medical charts of COVID-19 patients upon hospitalization for ICU and non-ICU-admitted patients. The *y*-axis; mean and standard deviation (SD).  ^*∗∗∗*^*p* < 0.002. ns, not significant.

**Figure 2:**
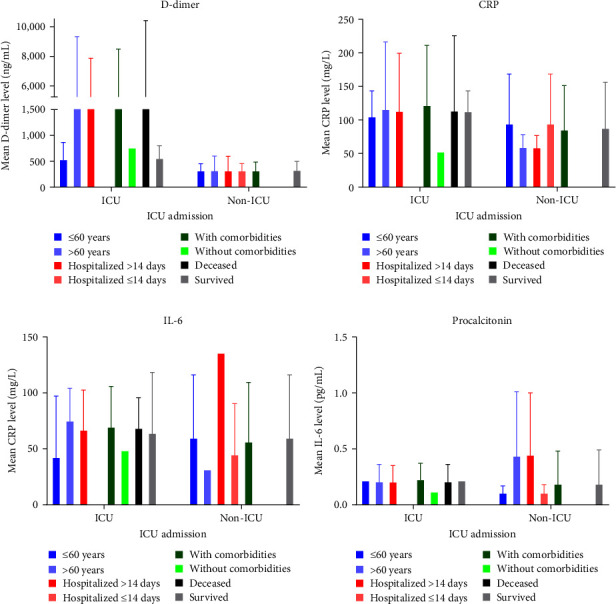
Impact of age, duration of hospitalization, and comorbidities on clinical parameters of COVID-19 patients following hospitalization. Data on D-dimer (a), CRP (b), IL-6 (c), and procalcitonin (d) were extracted from the medical charts of COVID-19 patients at presentation. The levels of these markers were compared among ICU and non-ICU admitted patients while stratifying for age, duration of hospitalization, presence or absence of comorbidities as well as survival. The *y*-axis represents the mean and standard deviation (SD). Blue bars represent age, red/pink bars represent the duration of hospitalization, green bars represent comorbidities, and black/grey bars represent mortality status.

Clinical laboratory biomarkers were available from medical records for 20 hospitalized patients (8 ICU and 12 non-ICU). At presentation, the median level of white blood cell (WBC) counts was significantly lower among patients in ICU (*p* = 0.0448) (Table 3). This may be due to viral erosion that results in direct inflammation and infection and consequently, excessive destruction of WBCs [36]. This is in agreement with previous studies reporting an association between decreased levels of WBCs in COVID-19 patients at presentation and disease severity and adverse outcomes [9, 37, 38]. Importantly, the mean level of D-dimer was significantly elevated among patients in ICU compared to those who did not require ICU admission (*p* = 0.0012); we did not detect any significant difference in the levels of CRP, IL-6, and PCT (Figure 1). Moreover, we detected significantly higher levels of D-dimer among deceased patients compared to those who survived (*p* = 0.003) (data not shown). When we stratified patients by ICU admission, we did not detect any significant difference in the levels of D-dimer, CRP, IL-6, and PCT based on age, duration of hospitalization, and the presence of comorbidities (Figure 2). Nevertheless, the mean level of D-dimer in ICU patients was higher compared to non-ICU patients reflecting the importance of D-dimer in predicting disease severity; the mean levels of CRP, IL-6, and PCT were similar among the two groups. The lack of significant difference might be attributed to our small sample size.

When comparing laboratory biomarkers at T0 and T1, our results showed that CRP levels were significantly higher at T0 in ICU and non-ICU patients (Table 4). When we stratified by age, CRP levels at T0 remained significantly higher than T1 in patients <60 years (*Z* = 2.667; *p* = 0.0076) and patients >60 years (*Z* = 2.429; *p* = 0.0152) (data not shown). Similarly, CRP levels at T0 were significantly higher among males (*Z* = 3.243; *p* = 0.0012). Moreover, CRP levels among patients with mild/moderate pneumonia and those with diffuse bilateral interstitial and airspace opacities were significantly higher at T0 compared to T1 (*Z* = 2.934; *p* = 0.0033 and *Z* = 2.903; *p* = 0.0037, respectively) (data not shown). This is expected since CRP is elevated following infection or inflammation, with higher levels indicating severe infection; the latter linked to hospitalization [39, 40]. When we stratified by patients who developed ARDS, we observed significantly higher levels of WBC counts and PCT at T1 compared to T0 (*Z* = −2.023; *p* = 0.043). However, we did not detect a significant difference in IL-6 levels. This is probably due to our small sample size.

**Figure 3:**
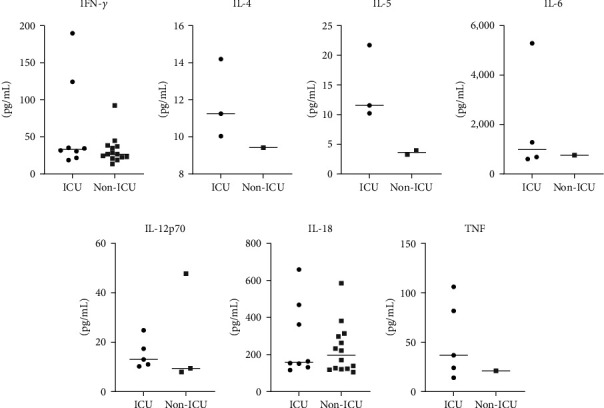
Levels of cytokines in ICU and non-ICU admitted patients. Sera from COVID-19 patients were collected to determine the levels of IFN-*γ* (a), IL-4 (b), IL-5 (c), IL-6 (d), IL-12p70 (e), IL-18 (f), and TNF (g). The horizontal line represents the median. The levels of IL-4, IL-5, and TNF were higher among ICU patients.

**Figure 4:**
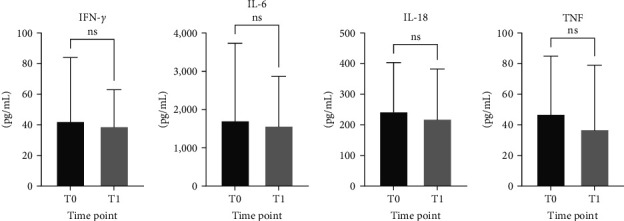
Levels of cytokines among hospitalized COVID-19 patients at presentation (T0) and time of discharge or death (T1). Sera from hospitalized COVID-19 patients were collected at T0 and T1, and levels of IFN-*γ* (a), IL-6 (b), IL-18 (c), and TNF (d) were measured. Data are shown as mean and standard deviation (SD). ns, not significant.

We compared the level of 11 cytokines (GM-CSF, IFN-*γ*, IL-1*β*, IL-2, IL-4, IL-5, IL-6, IL-12p70, IL-13, IL-18, and TNF) in hospitalized patients (8 ICU and 15 non-ICU admitted) at T0. We excluded GM-CSF, IL-1*β*, IL-2, and IL-13 during analysis since their values were OOR. Our data showed no significant difference when comparing the median of these cytokines between ICU and non-ICU admitted patients (Figure 3) despite the higher levels of IL-4, IL-5, and TNF detected among ICU-admitted patients. Interestingly, the median level of IL-12p70 was significantly higher in patients >60 years compared to those ≤60 years (*p* = 0.0209) regardless of ICU admission (data not shown). There was no significant difference when we compared cytokine levels with respect to the duration of hospitalization. Similarly, there was no significant difference between patients with and without comorbidities. Nevertheless, we observed higher IL-12p70 median levels among hypertensive compared to nonhypertensive patients though the difference was not statistically significant (data not shown). We did not detect any significant difference when we compared the mean levels of IFN-*γ*, IL-6, IL-18, and TNF between T0 and T1 (Figure 4), which might be attributed to our small sample size. We were unable to compare the levels of IL-4, IL-5, and IL-12p70 between T0 and T1 as their levels at T1 were OOR.

## 4. Discussion

COVID-19 remains a global public health threat despite the availability of effective vaccines. The continuous spread of SARS-CoV-2 is mainly driven by the emergence of new variants with immune-escape characteristics. Advanced age, male gender, and history of comorbid conditions (hypertension, diabetes, obesity, malignancy, kidney disease, cardiovascular disease (CVD), COPD, and chronic liver disease) are associated with severe disease (i.e., patients having severe dyspnea, extremely low oxygen saturation, respiratory distress, or requiring mechanical ventilation, ICU admission, or death) [4, 41, 42]. Our results are in agreement with previous studies assessing the clinical characteristics of hospitalized COVID-19 patients [43–45], whereby patients who required ICU admission in our cohort had higher median age than those who were not admitted to the ICU with hypertension and diabetes most commonly reported among all hospitalized patients. Moreover, COPD was associated with severe disease.

The assessment of laboratory biomarkers in COVID-19 patients is important in order to closely monitor the progression of illness and to guide treatment. The increase in the level of several biomarkers, including CRP, IL-6, as well as WBC counts, and D-dimer, has been associated with severe COVID-19 [46]. Importantly, D-dimer and CRP are considered important prognostic markers and predictors of COVID-19 severity and mortality [18, 25, 27, 28, 47–51]. Elevated D-dimer levels are most likely due to acute lung injury or the increased rate of thromboembolic complications among COVID-19 patients and thus the need for ICU admission [52]. Our results are aligned with previously reported data whereby high D-dimer levels were significantly associated with severe COVID-19 disease and mortality [39, 53–55]. Our results showed that CRP levels were significantly higher during hospitalization than at presentation in both groups of patients (ICU and non-ICU). Moreover, higher CRP levels were observed in ICU patients at presentation compared to non-ICU patients. This suggests that CRP increases during hospitalization and ICU admission of COVID-19 patients. This is in accordance with previously published data reporting CRP as an early indicator of COVID-19 disease progression and death [39, 40, 56].

The level of cytokines and immunologic dysregulation play a role in the severity of COVID-19 as well as multiple organ failure, respiratory distress syndrome, and septic shock [57, 58]. Hospitalized COVID-19 patients with ICU admission have higher plasma levels of IL-2, IL-7, IL-10, G-CSF, IP10, MCP-1, MIP-1A, and TNF compared to non-ICU patients [9]. Moreover, levels of IL-2, IL-2R, IL-4, IL-8, IFN-*γ*, and specifically IL-6 and IL-10 were significantly increased in severe compared to nonsevere cases [18, 51]. IL-6 is the primary trigger of CRS inducing the production of CRP by the liver; importantly, previous data showed a significant increase in IL-6 and CRP in severely ill patients [24, 37, 51] and thus predicted fatal outcomes [57]. In this study, we did not detect a significant difference in IL-6 levels (pro-inflammatory cytokine) between ICU and non-ICU admitted patients. This is in contrast to other reported data whereby the level of IL-6 was found to be higher in COVID-19 patients compared to healthy controls and was highly associated with disease severity and disease progression [59]. This is probably due to our small sample size. However, our data showed that the median level of IL-12p70, a pro-inflammatory cytokine, was significantly higher in older patients with severe illness compared to those with mild/moderate disease (≤60 years). This is consistent with previous studies reporting higher IL-12p70 levels in COVID-19 patients and those with severe outcomes [59–61]. Moreover, our results showed that IL-12p70 levels at presentation were higher among hypertensive compared to non-hypertensive COVID-19 patients, which is in line with previous data [60]. Limited data exist about the role of IL-12p70 in COVID-19. However, IL-12 is a heterodimeric cytokine activated by viral entry into the cells. Once activated, the pro-inflammatory cytokine IL-12 will activate other cytokines (such as IFN-*γ* and TNF) and immune cells involved in mediating inflammatory responses [60, 62]. Therefore, measuring the levels of IL-6, IL-12p70, CRP, and D-dimer at presentation can predict the risk of disease progression in COVID-19 patients.

Our study has several limitations. Our participants were recruited from a single tertiary hospital. Moreover, our study has a small sample size, and sera were not available for all participants for the measurement of cytokines. Importantly, this study lacks a control group of non-hospitalized COVID-19 patients. We were also unable to follow-up the patients longitudinally and measure their cytokine levels as well as laboratory biomarkers at multiple time points during hospitalization and following discharge. This is important in order to monitor the trends of biomarkers and cytokines between patients with mild/moderate and severe illness, determine the peak level of each cytokine and biomarker during hospitalization and correlate them with severe and fatal outcomes.

## 5. Conclusion

The CRS described in COVID-19 patients with severe outcomes results in deleterious clinical manifestations that might lead to death. There is a lack of studies on cytokines and clinical characteristics of hospitalized COVID-19 patients in Lebanon. Consequently, more studies are needed to assess the kinetics of cytokines in these patients and their clinical impact on organ systems as well as on the course of treatment. Moreover, more studies are needed to longitudinally assess cytokine levels in COVID-19 patients and correlate them with postacute COVID-19 syndrome. The latter is increasingly being reported in a large portion of COVID-19 patients 3–12 months following recovery from the acute phase of illness [63].

## Figures and Tables

**Table 1:** Demographics and clinical characteristics of study participants.

Variables	*n*	%
Age (years) (*N* = 51)		
18–40	8	15.7
41–60	18	35.3
>60	25	49
Gender (*N* = 51)		
Male	37	72.5
Female	14	27.5
Comorbidities (*N* = 51)		
Yes	41	80.4
No	10	19.6
Comorbid conditions (*N* = 41)		
Hypertension	29	70.7
Diabetes	16	39
Dyslipidemia	16	39
CVD	13	31.7
Cancer	13	31.7
Kidney disease	9	22
COPD	5	12.2
COVID-19 vaccination status (*n* = 51)		
Yes	4	8
No	36	70.5
Not available	11	21.5
Duration of hospitalization (*N* = 51)		
≤14 days	38	74.5
>14 days	13	25.5
ICU admission (*N* = 51)		
Yes	11	21.5
No	40	78.5
Length of ICU stay (*N* = 11)		
≤14 days	4	36.4
>14 days	7	63.6
Supplementary oxygen requirement (*N* = 50)		
Yes	35	70
No	15	30
Mechanical ventilation (*N* = 49)		
Yes	6	12.2
No	43	87.8

Treatment		

CRS treatment (*N* = 51)		
Yes	12	23.5
No	39	76.5
Anti-inflammatory treatment (*N* = 50)		
Yes	18	36
No	32	64
Corticosteroid treatment (*N* = 50)		
Yes	31	62
No	19	38
Ivermectin (*N* = 51)		
Yes	20	39.2
No	31	60.8
Remdesivir (*N* = 50)		
Yes	28	56
No	22	44
Anticoagulant treatment (*N* = 51)		
Yes	45	88.2
No	6	11.8
IL-6 receptor antagonist (*N* = 50)		
Yes	12	24
No	38	76
Vasodilation treatment (*N* = 50)		
Yes	2	4
No	48	96
Cardiomyopathy treatment (*N* = 50)		
Yes	1	2
No	49	98
Deceased (*N* = 49)		
Yes	9	18.4
No	40	81.6

ICU, intensive care unit; CRS, cytokine release syndrome; IL-6, interleukin-6.

**Table 2:** Relationship between patient characteristics and ICU admission.

	Non-ICU	ICU	*p*-Value^a^
Median age (range) (years)	57.5 (26–90)	70 (24–89)	0.0587
Gender			**0.023**
Male	26 (65.0%)	11 (100.0%)	
Female	14 (35.0%)	0 (0.0%)	
Smoking status			0.9
Current smoker	8 (22.2%)	3 (30.0%)	
Former smoker	9 (25.0%)	2 (20.0%)	
Never smoker	19 (52.8%)	5 (50.0%)	
Comorbidities			
Hypertension	24 (60.0%)	5 (45.5%)	0.498
CVD	11 (27.5%)	2 (18.2%)	0.706
Diabetes	13 (32.5%)	3 (27.3%)	1
Dyslipidemia	12 (30.0%)	4 (36.4%)	0.723
Cancer	9 (22.5%)	4 (36.4%)	0.439
Kidney disease	7 (17.5%)	2 (18.2%)	1
Asthma	5 (12.5%)	0 (0.0%)	0.572
COPD	1 (2.5%)	4 (36.4%)	**0.006**
Oxygen requirement and mechanical ventilation			
Oxygen requirement	24 (61.5%)	11 (100.0%)	**0.021**
Median duration of oxygen requirement (range) (days)	6 (2–22)	15 (1–68)	0.1662
Mechanical ventilation	0 (0.0%)	6 (60.0%)	**<0.001**
Median duration of mechanical ventilation (range) (days)	–	24 (15–54)	-
Treatment			
CRS	5 (12.5%)	7 (63.6%)	**<0.001**
Anti-inflammatory	16 (41.0%)	2 (18.2%)	0.287
Corticosteroids	22 (56.4%)	9 (81.8%)	0.17
Ivermectin	16 (40.0%)	4 (36.4%)	1
Remdesivir	21 (53.8%)	7 (63.6%)	0.734
Septic shock treatment	0 (0.0%)	6 (54.6%)	**<0.001**
Anticoagulant treatment	35 (87.5%)	10 (90.9%)	1
IL-6 receptor antagonist	6 (15.4%)	6 (54.6%)	**0.014**
Vasodilation treatment	0 (0.0%)	2 (18.2%)	**0.045**
Cardiopulmonary treatment	0 (0.0%)	1 (10.0%)	0.2
Deceased	3 (7.9%)	6 (54.6%)	**0.002**

CVD, cardiovascular disease; COPD, chronic obstructive pulmonary disease; CRS, cytokine release syndrome. ^a^Fisher's exact test or Mann–Whitney *U* test. Values in bold indicate significant *p* values (*p* < 0.05).

**Table 3:** Clinical laboratory parameters upon presentation among study participants.

Biomarker (normal range)	ICU (*n* = 8)	Non-ICU (*n* = 12)	*p*-Value^*∗*^
RBC (4.5–6.5 × 10^6^/mm^3^)	4.4	4.6	0.3957
WBC (4–11 × 10^3^/mm^3^)	5.3	6.45	**0.0448**
Platelets (150–400 × 10^3^/mm^3^)	173.5	235.5	0.1425
Neutrophils (40–65%)	82.5	84.5	0.7282
HGB (males 13–18 g/dL) (females 12–15 g/dL)	12.5	13	0.8163

ICU, intensive care unit; RBC, red blood cells; WBC, white blood cells.  ^*∗*^Mann–Whitney *U* test. Values in bold indicate significant *p* values (*p* < 0.05).

**Table 4:** Clinical biomarkers at T0 and T1 among ICU and non-ICU patients.

Variable	Group	Negative ranks		Positive ranks		Test statistics	
		*n*	Sum of ranks	*n*	Sum of ranks	*Z*	*P* ^*∗*^
WBC (10^3^/mm^3^)	ICU	8	36	0	0	−2.521	**0.0117**
	Non-ICU	4	26	8	52	1.02	0.3076

Neutrophils (%)	ICU	5	20	3	16	−0.28	0.7794
	Non-ICU	2	6.5	10	71.5	2.55	**0.0108**

Platelets (10^3^/mm^3^)	ICU	5	26	3	10	−1.12	0.2626
	Non-ICU	7	63	5	15	−1.883	0.0597

Procalcitonin (ng/mL)	ICU	5	18	1	3	−1.572	0.1159
	Non-ICU	1	6	7	38	1.899	0.0576

D-dimer (ng/mL)	ICU	7	28	1	8	−1.4	0.1614
	Non-ICU	5	19	4	26	0.415	0.6784

CRP (mg/L)	ICU	1	2	7	34	2.24	**0.0251**
	Non-ICU	1	3	11	75	2.824	**0.0047**

IL-6 (pg/mL)	ICU	2	3	0	0	−1.342	0.1797
	Non-ICU	0	0	4	10	1.826	0.0679

WBC, white blood cells; CRP, C-reactive protein; IL-6, interleukin 6.  ^*∗*^Wilcoxon signed rank test. Values in bold indicate significant *p* values (*p* < 0.05).

## Data Availability

The clinical, biological, and demographic data used to support the findings of this study are included within the article.
